# The impact of the ‘Better Care Better Value’ prescribing policy on the utilisation of angiotensin-converting enzyme inhibitors and angiotensin receptor blockers for treating hypertension in the UK primary care setting: longitudinal quasi-experimental design

**DOI:** 10.1186/s12913-015-1013-y

**Published:** 2015-09-10

**Authors:** Amanj Baker, Li-Chia Chen, Rachel A. Elliott, Brian Godman

**Affiliations:** Division for Social Research in Medicines and Health, School of Pharmacy, University of Nottingham, East Drive, University Park, Nottingham, NG7 2RD UK; Department of Pharmacology, College of Pharmacy, Hawler Medical University, Erbil, Iraq; Division of Clinical Pharmacology, Karolinska Institute, Karolinska University Hospital, Huddinge, Stockholm Sweden; Strathclyde Institute of Pharmacy and Biomedical Sciences, Strathclyde University, Glasgow, UK

## Abstract

**Background:**

In April/2009, the UK National Health Service initiated four Better Care Better Value (BCBV) prescribing indicators, one of which encouraged the prescribing of cheaper angiotensin-converting enzyme inhibitors (ACEIs) instead of expensive angiotensin receptor blockers (ARBs), with 80 % ACEIs/20 % ARBs as a proposed, and achievable target. The policy was intended to save costs without affecting patient outcomes. However, little is known about the actual impact of the BCBV indicator on ACEIs/ARBs utilisation and cost-savings. Therefore, this study aimed to evaluate the impact of BCBV policy on ACEIs/ARBs utilisation and cost-savings, including exploration of regional variations of the policy’s impact.

**Methods:**

This cross-sectional study used data from the UK Clinical Practice Research Datalink. Segmented time-series analysis was applied to monthly ACEIs prescription proportion, adjusted number of ACEIs/ARBs prescriptions and costs.

**Results:**

Overall, the proportion of ACEIs prescription decreased during the study period from 71.2 % in April/2006 to 70.7 % in March/2012, with a small but a statistically significant pre-policy reduction in its monthly trend of 0.02 % (*p* < 0.001). Instantly after its initiation, the policy was associated with a sudden reduction in the proportion of ACEIs prescription; however, it resulted in a statistically significant increase in the post-policy monthly trend of ACEIs prescription proportion of 0.013 % (*p* < 0.001), resulting in an overall post-policy slope of −0.007 %. Despite this post-policy induced increment, the policy failed to achieve the 80 % target, which resulted in missing a potential cost-saving opportunity. The pre-policy trend of the adjusted number of ACEIs/ARBs prescriptions was increasing; however, their trends declined after the policy implementation. The policy affected neither total ACEIs/ARBs cost nor individual ACEIs or ARBs costs.

**Conclusions:**

ACEIs/ARBs utilisation was not affected by the BCBV policy. The small increase in post-policy ACEIs prescription proportion was not associated with any savings. This study represents a case study of a failed or ineffective policy and thus provides key learning lessons for other healthcare authorities. Given the existing opportunity of potential cost-savings from achieving the 80 % target, specific measures would be needed to enhance the policy implementation and uptake; however, this must be balanced against other cost-saving policies in other high-priority areas.

**Electronic supplementary material:**

The online version of this article (doi:10.1186/s12913-015-1013-y) contains supplementary material, which is available to authorized users.

## Background

Hypertension is the leading cause of cardiovascular disease, resulting in a substantial healthcare burden globally [1]. The National Institute for Health and Care Excellence (NICE) reported that at least one-quarter of adults in the UK had primary hypertension in 2011 [2]. Drugs affecting the renin-angiotensin system (RAS), including angiotensin-converting enzyme inhibitors (ACEIs) and angiotensin receptor blockers (ARBs), are among the most frequently prescribed antihypertensive drugs [3].

Over the past decade, there has been a marked increase in the utilisation and cost of RAS agents, especially ARBs [4–7]. In the UK, the utilisation of ACEIs/ARBs increased after the implementation of the Quality and Outcomes Framework (QoF) in April 2004 and the publication of revised NICE guidelines for hypertension management in June 2006 [8]. With GB£277 million spent on ARBs in England in 2009, ARBs represented the fourth highest drug cost in the National Health Service (NHS England) [9]. Whereas in Scotland, approximately GB£26.27 million were spent on ARBs, making them the seventh most expensive drug class [10]. The NHS Business Services Authority reported a 66 % increase in the prescription items for ACEIs/ARBs to 13.4 million items in primary care in England, over a five-year period from June/2004 to June/2009 [11]. Furthermore, the Prescription Cost Analysis (PCA) of England-2011, issued by the Health and Social Care Information Centre of the UK, considered ACEIs/ARBs as being the second most commonly prescribed drug class in English primary care, accounting for 6 % of all prescribed drugs [12].

ACEIs and ARBs are considered to have equal efficacy in hypertension control and cardiovascular disease prevention [3, 13], except that ACEIs are more likely to cause a dry cough (2–10 %) [3] than ARBs. However, only 2–3 % of patients actually stopped ACEIs in clinical trials due to dry cough [13, 14]. Therefore, efficient prescribing of RAS agents, i.e., prescribing cheaper generic ACEIs instead of more expensive patent protected ARBs, is considered an important cost-saving strategy.

The efficient prescribing of RAS agents has been under intensive scrutiny in many European countries and regions, ex. Austria, Croatia, Serbia, Scotland, Spain, Sweden, and the Republic of Srpska, with multiple initiatives targeting ACEIs/ARBs prescribing to enhance their efficient use, including prescriber education, prescribing targets and restrictions, and financial incentives [15–17]. In the UK, the NHS Institute for Innovation and Improvement released four Better Care Better Value (BCBV) prescribing indicators in April 2009 [18], one of which targeted the ACEIs prescribed as a proportion of RAS agents prescribed overall. This built on initiatives via NICE, National Prescribing Centre (NPC) and Primary Care Trusts (PCTs) to encourage the prescribing of generic ACEIs first line if a RAS agent was being considered, with ARBs reserved for patients intolerant to ACEIs [19–21].

The BCBV policy was passively disseminated through emails to people who are responsible for prescribing and medicine management within NHS organisations, discussion in workshops and/or via emails to individual GPs informing them about the establishment of the policy with a link to the full policy’s details. However, the policy was neither linked to any central financial incentives, legislation enforcement nor any other strategies to increase its uptake, although there could have been local PCT initiatives. Although a clear target for ACEIs prescription proportion was not specified, a proportion of at least 80 % ACEIs had been proposed by the NICE based on experts’ opinions [2] and was considered as an achievable target [22]. The UK Office of Fair Trading (OFT) [23] suggested a higher target of 95 % ACEIs based on the fact that only 2–3 % of patients in clinical trials actually stopped ACEIs due to dry cough [24, 25] and the OFT panel opinion that only about 5 % of patients would stop ACEIs therapy due to dry cough [23]. However, the 80 % target seems more likely to be acceptable as it would leave room for preserving physicians’ and patients’ choice and autonomy. For the BCBV indicator to achieve its goal, this required general practitioners (GPs) to initiate new patients on ACEIs and switch existing ARBs users to ACEIs when appropriate.

In the UK, GPs act as a gatekeeper to the NHS and under the new NHS structure of Clinical Commissioning Groups (CCGs), they are in charge of arranging the health services that meet the local needs through working with patients and health and social care partners. Consequently, GPs have greater freedom and leadership roles [26].

The importance of the appropriate prescribing of RAS drugs in saving the NHS money has been further emphasised by the Quality, Innovation, Productivity and Prevention (QIPP) programme established in 2010, which aimed to enhance the value derived from NHS money while maintaining or improving quality of provided care through the optimisation of medicines use [27]. This emphasis has been performed by identifying and including RAS drugs as one of the key topic areas, where maintaining or improving quality while improving the value of money is a potential opportunity, in the QIPP key therapeutic topics document, which summarises the evidence on the topics identified to support the QIPP programme [28].

The BCBV policy intended to improve the efficient use of healthcare resources and quality of care within NHS organisations, and compares the ACEIs/ARBs utilisation across organisations [29], which was shown to vary across the UK regions [22, 30]. The policy was expected to achieve a marked cost-saving to the NHS, which can be invested to support other areas within NHS such as funding new premium priced innovative medicines. Within the NHS, medicines represent one of the greatest non-salary areas of expenditure and have been scrutinised intensively for many years [31].

Inappropriate spending on prescribed medicines by GPs in 1994 was estimated to cost about GB£300 million a year [32]. Even more than a decade later, the National Audit Office (NAO) report concerning prescribing costs in primary care in the UK in 2007 [33] found that a potential cost saving of more than GB£200 million a year could be achieved, without compromising the quality of care, through efficient prescribing in four therapeutic drug classes, including RAS drugs. In fact, this estimated cost-saving figure was the essence behind initiating the BCBV prescribing indicator.

Furthermore, the National Prescribing Centre of England estimated a potential annual future saving ranging from GB£68 million to GB£149 million if the ACEIs prescription proportion increased from 70 % in 2009 to 80 % and 90 %, respectively [22]. However, currently it is also recognised that this potential saving could have been reduced by the concurrent introduction of several relatively lower cost generic ARBs (losartan, candesartan, irbesartan, valsartan). Apart from the NAO estimated cost saving figures from efficient prescribing of RAS drugs, little is known about the actual impact of BCBV prescribing indicator on ACEIs/ARBs utilisation and cost-savings.

### Aim and objectives

This study aimed to evaluate the impact of the BCBV prescribing policy on ACEIs/ARBs utilisation and cost-saving for treating hypertension in the UK primary care setting and to explore potential regional variations of the policy’s impact.

## Methods

### Study design and data sources

This cross-sectional study adopted a natural quasi-experimental design [34] and applied a segmented time-series analysis [35] to investigate the impact of BCBV prescribing policy on ACEIs/ARBs utilisation, using data from the UK Clinical Practice Research Datalink (CPRD) database [36] from April 2006 to March 2012. This study protocol was approved by the Independent Scientific Advisory Committee for study quality standard and access of CPRD database (protocol number 13_150).

The CPRD contains anonymised longitudinal clinical data collected from GPs’ daily records in primary care. It is broadly representative of the UK population in terms of patient and practice characteristics [37] and covers about 8.5 % of the UK population. In March 2015, it contained longitudinal clinical records of more than 13.7 million patients and 5.4 million active patients from 685 primary care practices across the UK [38].

CPRD is a prescribing dataset containing detailed information about prescriptions issued by GPs. Drug prescription information is well recorded and can be linked to an individual patient's clinical and medical information. Therefore, CPRD is an optimal data source for analysing drug utilisation in the UK primary care, where there is currently no means to collect patient identity data across the whole population, particularly in evaluating the impact of BCBV indicator as it provides comprehensive information about GPs’ prescribing habits which the policy aimed to make more efficient.

Despite all those merits, CPRD data were available up to March 2012 at the time of conducting this study. Consequently, data from the Prescription Cost Analysis (PCA) of England [39] was used to estimate the potential up to date national cost-saving impact of this BCBV policy. Furthermore, to ensure that CPRD data reflected the national data, data from the Health and Social Care Information Centre (HSCIC) [40] was used to validate the study results. Both PCA and HSCIC datasets contain aggregate level information about all the prescriptions that dispensed in the community in England; however, they lack clinical information such as the indication of use as well as does not allow to link prescriptions to an individual patient.

This study included all antihypertensive prescriptions issued for adults (≥18 years old) with primary hypertension from April 2006 to March 2012. As the policy implementation involves both actions of starting incident patients on ACEIs and switching prevalent ARBs users to ACEIs, both incident and prevalent patients were included to obtain a full picture about the policy impact. Patients with primary hypertension and their antihypertensive prescriptions were identified using related diagnostic Read codes and product codes, respectively.

### Utilisation measures

The proportion of ACEIs prescriptions was calculated monthly as the primary utilisation measure, and was stratified by the 13 UK regions because we expected potential variations in the policy adoption in various regions due to the lack of central incentives to implement the policy. The number of ACEIs/ARBs prescriptions and ACEIs/ARBs prescription costs were repeatedly measured for each month. The total number of patients with primary hypertension and total number of antihypertensive prescriptions were also calculated and stratified by the 13 regions. Both single strength ACEIs/ARBs and fixed-dose combinations (FDCs) of ACEIs/ARBs with other antihypertensive drugs were included in the analysis, although previous UK studies have shown that FDCs only accounted for less than 2 % of total RAS drugs [10, 15].

The number of prescriptions was used as the utilisation measure for ACEIs and ARBs as opposed to the internationally recognised defined daily doses [41] because the number of prescriptions has been the utilisation metric used by UK NHS professionals to calculate BCBV indicators [42]. To ensure that the observed changes in the number of prescriptions were not an artefact of monthly alteration in both number of active patients registered in CPRD and number of patients with hypertension, the number of prescriptions was transformed and presented as adjusted monthly number of prescriptions which was derived from dividing the absolute number of prescribed prescriptions by the prevalence of hypertension in the same month, i.e., the resulted number, therefore represents the number of prescriptions per one-percent hypertension prevalence. Monthly hypertension prevalence was calculated by dividing the number of hypertensive patients by the number of active patients registered in CPRD in the same month.

Prescription costs were calculated by multiplying prescribed quantities of ACEIs and ARBs by unit prices obtained from the British National Formulary (BNF), March 2012 [43]. The 2012 unit price was used rather than historical prices in order to estimate the change in drug expenditure from the NHS perspective, whilst controlling for inflation [44], as well as because March 2012 was the last available date for CPRD data at the time of the study.

### Prediction of the potential cost saving of BCBV policy

The potential cost-saving in March 2012, had the 80 % ACEIs target been achieved was estimated, which was then applied to the national ACEIs/ARBs costing figure, obtained from England Prescription Cost Analysis (PCA) March 2012 data [39], to estimate the potential cost-saving on a national level.

Firstly, the predicted number of ACEIs prescriptions was calculated by multiplying the actual total number of ACEIs/ARBs prescriptions in March 2012 by 80 %, which was afterwards subtracted from actual total ACEIs/ARBs prescriptions to calculate the predicted number of ARBs prescription. Secondly, the predicted number of ACEIs and ARBs prescriptions was multiplied by the mean cost of ACEIs and ARBs per prescription in March 2012, respectively, to calculate the predicted cost of ACEIs and ARBs prescriptions. The mean cost of single ACEIs and ARBs prescription were estimated by dividing the actual total cost of ACEIs and ARBs by their actual number of prescriptions in March 2012, respectively. Finally, the potential cost saving was calculated by subtracting total actual ACEIs/ARBs cost in March 2012 from the total predicated ACEIs/ARBs cost.

Data from PCA of England in June 2014 [39], the most up-to-date data available at the date of submission, which contains the number of dispensed antihypertensive drug prescriptions and their costs, were also analysed to estimate the impact of the availability of low cost generic ARBs on the potential cost-saving of BCBV policy if the 80 % target had been achieved in June 2014 in England. The potential cost-saving was estimated using the same procedures described above, but applying the 2014 costs.

### Data analysis

Segmented regression of the interrupted time series [35] was used to analyse the time-series of monthly utilisation measures from April 2006 to March 2012. The analysis of ACEIs prescription proportion was stratified by the 13 UK regions. As the BCBV indicator targeted the proportion of ACEIs prescribed as a % of total RAS prescriptions, prescriptions were used as the unit of analysis and thus prescription level analysis was performed.

The trends of utilisation (β_1_) in the pre-intervention period, and any changes in the levels (β_2_) and trends of utilisation (β_3_) following the intervention were assessed and presented [35]. To control for potential confounders [34], the fitted models for all utilisation measures were adjusted for the launch of generic perindopril in October 2007 and the launch of generic losartan in July 2010. Data analysis was performed using STATA 12 (StataCorp, Texas, USA).

The following segmented regression model was fitted for each individual study outcome measure:

$$ \begin{array}{l}{\mathit{\mathsf{Y}}}_{\mathit{\mathsf{t}}} = {\mathit{\mathsf{\beta}}}_{\boldsymbol{\mathsf{0}}} + {\boldsymbol{\mathsf{\boldsymbol{\beta}}}}_{\boldsymbol{\mathsf{1}}}*\mathit{\mathsf{t}}\mathit{\mathsf{i}}\mathit{\mathsf{m}}e + {\boldsymbol{\mathsf{\boldsymbol{\beta}}}}_{\boldsymbol{\mathsf{2}}}*\mathit{\mathsf{BCBV}}\ \mathit{\mathsf{i}}n\mathit{\mathsf{t}}\boldsymbol{e}\mathit{\mathsf{r}}\mathit{\mathsf{v}}en\mathit{\mathsf{t}}\mathit{\mathsf{i}}\mathit{\mathsf{o}}n + {\boldsymbol{\mathsf{\boldsymbol{\beta}}}}_{\boldsymbol{\mathsf{3}}}*\mathit{\mathsf{t}}\mathit{\mathsf{i}}\mathit{\mathsf{m}}e\ \mathit{\mathsf{a}\mathsf{f}\mathsf{t}}\boldsymbol{e}\mathit{\mathsf{r}}\ \mathit{\mathsf{BCBV}}\ \mathit{\mathsf{i}}n\mathit{\mathsf{t}}e\mathit{\mathsf{r}}\mathit{\mathsf{v}}en\mathit{\mathsf{t}}\mathit{\mathsf{i}}\mathit{\mathsf{o}}n + {\boldsymbol{\mathsf{\boldsymbol{\beta}}}}_{\boldsymbol{\mathsf{4}}}*\\ {}\mathit{\mathsf{l}}\mathit{\mathsf{a}}\mathit{\mathsf{u}}n\mathit{\mathsf{c}}\mathit{\mathsf{h}}\ \mathit{\mathsf{o}\mathsf{f}}\ \mathit{\mathsf{g}}ene\mathit{\mathsf{r}}\mathit{\mathsf{i}}\mathit{\mathsf{c}}\ \mathit{\mathsf{l}\mathsf{osarta}}n + {\mathit{\mathsf{\beta}}}_{\mathit{\mathsf{5}}}*\mathit{\mathsf{t}}\mathit{\mathsf{i}}\mathit{\mathsf{m}}e\ \mathit{\mathsf{a}\mathsf{f}\mathsf{t}}\boldsymbol{e}\mathit{\mathsf{r}}\ \mathit{\mathsf{l}}\mathit{\mathsf{a}}\mathit{\mathsf{u}}n\mathit{\mathsf{c}}\mathit{\mathsf{h}}\ \mathit{\mathsf{o}\mathsf{f}}\ \mathit{\mathsf{g}}ene\mathit{\mathsf{r}}\mathit{\mathsf{i}}\mathit{\mathsf{c}}\ \mathit{\mathsf{l}\mathsf{osarta}}n + {\mathit{\mathsf{\beta}}}_{\mathit{\mathsf{6}}}*\mathit{\mathsf{l}}\mathit{\mathsf{a}}\mathit{\mathsf{u}}n\mathit{\mathsf{c}}\mathit{\mathsf{h}}\ \\ {}\mathit{\mathsf{o}\mathsf{f}}\ \mathit{\mathsf{g}}\boldsymbol{e}n\boldsymbol{e}\mathit{\mathsf{r}}\mathit{\mathsf{i}}\mathit{\mathsf{c}}\ \mathit{\mathsf{p}}e\mathit{\mathsf{r}}\mathit{\mathsf{i}}n\mathit{\mathsf{dopril}} + {\boldsymbol{\mathsf{\boldsymbol{\beta}}}}_{\boldsymbol{\mathsf{7}}}*\mathit{\mathsf{t}}\mathit{\mathsf{i}}\mathit{\mathsf{m}}e\ \mathit{\mathsf{a}\mathsf{f}\mathsf{t}}e\mathit{\mathsf{r}}\ \mathit{\mathsf{l}}\mathit{\mathsf{a}}\mathit{\mathsf{u}}n\mathit{\mathsf{c}}\mathit{\mathsf{h}}\ \mathit{\mathsf{o}\mathsf{f}}\ \mathit{\mathsf{g}}ene\mathit{\mathsf{r}}\mathit{\mathsf{i}}\mathit{\mathsf{c}}\ \mathit{\mathsf{p}}e\mathit{\mathsf{r}}\mathit{\mathsf{i}}n\mathit{\mathsf{dopril}} + {e}_{\mathit{\mathsf{t}}}\end{array} $$

*Y*_*t*_ is the monthly outcome measure. *Time* is a continuous variable referring to time, in months, from the start of the observation period, range from 1 to 72 from the start to end of the study period. *BCBV intervention* is a binary variable, takes the value of zero and one for the time before and after intervention implementation, respectively, i.e., zero from the start till month 36, then one afterward. *Time after BCBV intervention* is a continuous variable counting the number of months after the BCBV intervention at time t, coded 0 before the intervention and (time-36) after the intervention. *Launch of generic losartan* is a binary variable, takes the value of zero and one for the time before and after intervention implementation, respectively, i.e., zero from the start till month 51, then one afterward. *Time after launch of generic losartan* is a continuous variable counting the number of months after the launch of generic losartan at time t, coded 0 before the intervention and (time-51) after the intervention. *Launch of generic perindopril* is a binary variable, takes the value of zero and one for time before and after intervention implementation, respectively, i.e., zero from the start till month 18, then one afterward. *Time after launch of generic perindopril* is a continuous variable counting the number of months after the launch of generic perindopril at time t, coded 0 before the intervention and (time-18) after the intervention. *e*_*t*_ is an error term at the time t which describes the random variability in outcome not explained by the model.

To obtain unbiased estimates for the intervention effect, the final fitted models were checked for autocorrelation in the residuals using an autocorrelation function graph [35] and the Portmanteau test [45], and any auto-correlation was adjusted for using Prais-Winsten regression and an auto-regressive integrated moving-average model [34]. The regression coefficients with 95 % confidence intervals were presented for the most parsimonious model by excluding non-significant variables (*p* > 0.05) using the stepwise backward elimination method [35]. However, all the parameter estimates with their 95 % confidence intervals were also presented as additional files.

Since the baseline ACEIs prescription proportion before the implementation of BCBV policy differed in various regions [22], the results of ACEIs prescription proportions analysis for the 13 UK regions were grouped into high (>74 %), intermediate (65 %-74 %) and low levels (<65 %) groups, based on the ACEIs prescription proportion in April 2006.

Ideally, to separate the effect of BCBV policy from other policies that might have been established at the same time, the impact of BCBV policy on ACEIs/ARBs utilisation should be compared to those in a BCBV policy-free group (reference group). However, it was not possible to identify a BCBV policy-free group as the policy was implemented nationally. As an alternative, the utilisation of the other four major antihypertensive drug classes, i.e., diuretics, calcium-channel blockers (CCBs), beta-blockers (BBs), and ‘Others’ (including vasodilators, centrally acting drugs, alpha-blockers), that should not be affected by this specific policy were used as reference (policy-free) groups [34, 35]. With this approach, separate models were re-specified with the utilisation of the other four antihypertensive drug classes as the dependent variable in each instance.

To validate the study results, the quarterly trends in the number of ACEIs/ARBs prescriptions and other antihypertensive drug classes were also compared with the quarterly England dispensing data, which contains the number of antihypertensive drugs prescriptions dispensed in the primary care setting in England for treatment of all conditions, and obtained directly from the Health and Social Care Information Centre (HSCIC) [40].

## Results

### Adjusted monthly number of antihypertensive prescriptions

**Fig. 1:**
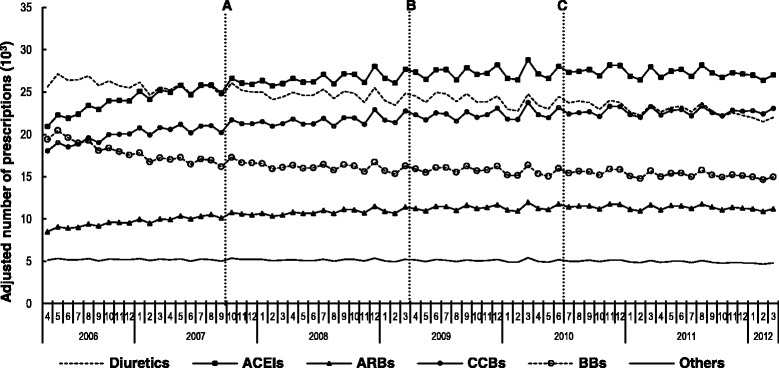
Adjusted monthly number of prescriptions for the six antihypertensive drug classes. **a** time point when generic perindopril became available (October 2007); **b** time point when BCBV policy was implemented April 2009); **c** time point when generic losartan launched (July 2010); ACEIs: Angiotensin converting enzyme inhibitors; ARBs: Angiotensin receptor blockers; CCBs: Calcium channel blockers; BBs: Beta-blockers

Overall, 44,408,931 antihypertensive prescriptions were issued to 617,334 patients with primary hypertension during the study period. On average, each patient had one antihypertensive drug prescription per month during the six-year study period, with a median duration of 28 days for the individual antihypertensive drug classes, which is consistent with the normal practice of prescribing one-month supply in the UK. ACEIs were the most frequently prescribed antihypertensive drugs with 11,222,597 (25.3 %) prescriptions, followed by diuretics (*n* = 10,297,565, 23.3 %), CCBs (*n* = 9,245,498, 20.8 %), BBs (*n* = 6,873,068, 15.5 %), ARBs (*n* = 4,610,122, 10.4 %), and “Others” (*n* = 2,160,081, 4.9 %). In April 2006, diuretics were the most frequently prescribed antihypertensive class (Fig. 1). In total, the adjusted number of ACEIs, ARBs, and CCBs prescriptions showed an absolute increase during the study period of 4.8 %, 2.2 %, and 3.9 %, respectively. Consequently, ACEIs became the leading prescribed antihypertensive drug category by the end of the study period.

Prior to the BCBV policy, there was a significant increase in baseline trend in the adjusted number of prescriptions of ACEIs (β_1_: 135.7, *p* < 0.001), ARBs (β_1_: 65.9 %, *p* < 0.001), CCBs (β_1_: 90.4, *p* < 0.001) (Table 1), but significant reduction in diuretics (β_1_: −55.4, *p* < 0.001), BBs (β_1_: −102.7, *p* < 0.001) and “Others” (β_1_: −3.0, *p* < 0.001). Although the implementation of the BCBV policy had no significant effect on the level of the adjusted number of prescriptions (β_2_) for all drug classes, there was a significant reduction in the trend of the adjusted number of prescriptions (β_3_) for all drug classes thereafter, except for diuretics (Table 1). All the observed changes were small in magnitude, albeit statistically significant. All the parameter estimates were presented in Additional file 1.

**Table 1:** Segmented regression analysis on monthly adjusted number of prescription of the six antihypertensive drug classes

Variables	β_1_^(a)^	β_2_^(b)^	β_3_^(c)^	β_4_^(d)^	β_5_^(e)^	β_6_^(f)^	β_7_^(g)^
ACEIs prescription proportion (%)	−0.02 (−0.2, −0.01)	−0.30 (−0.44, −0.16)	0.013 (0.007, 0.02)	---	---	---	---
Adjusted number of prescriptions							
ACEIs	135.7 (117.8, 153.6)	---	−149.9 (−181.4, −118.4)	---	---	---	---
ARBs	65.9 (58.7, 72.0)	---	−67.2 (−79.8, −54.6)	---	---	---	---
Diuretics	−55.4 (−61.9, −48.9)	---	---	---	---	---	---
CCBs	90.4 (77.0, 103.9)	---	−77.4 (−101.1, −53.8)	---	---	---	---
BBs	−102.7 (−119.0, −86.5)	---	−75.3 (−98.3, −52.4)	---	---	---	---
“Others”	−3.0 (−4.9, −1.0)	---	−15.6 (−21.1, −10.1)	---	---	---	---

Regression coefficients (95 % confidence intervals) for the final model (the most parsimonious models); ^**(a)**^baseline trend; ^**(b)**^level change following BCBV policy; ^**(c)**^trend change following BCBV policy; ^**(d)**^level change following generic losartan availability; ^**(e)**^trend change following generic losartan availability; ^**(f)**^level change following generic perindopril availability; ^(g)^trend change following generic perindopril availability; −--: indicates insignificant estimates at 0.05 level, after stepwise backward elimination; ACEIs: Angiotensin converting enzyme inhibitors; ARBs: Angiotensin receptor blockers; CCBs: Calcium channel blockers; BBs: Beta-blocker

### ACEIs prescription proportion

The proportion of monthly ACEIs prescription was 71.2 % in April 2006, but had declined to 70.7 % in March 2012. There was a significant reduction in the monthly trend of ACEIs prescription proportion (β_1_: −0.02 %, *p* < 0.001) before the implementation of BCBV policy, and the level of ACEIs prescription proportion further reduced when the policy was implemented (β_2_: −0.3 %, *p* < 0.001). However, the policy resulted in a sustained significant increase in the monthly ACEIs prescription trend (β_3_: 0.013 %, *p* < 0.001) thereafter (Table 1), i.e., the policy reversed the existing declining monthly trend by 0.013 %, so that the slope of the post-policy line (β_1_+ β_3_) was declining by 0.007 % compared with the pre-policy declining rate of 0.02 %. Although all the observed changes were very minute in magnitude, they were statistically significant.

Seven of the 13 regions’ baseline ACEIs prescription proportion ranged from 65 to 74 % of total RAS prescriptions (Table 2), while four and two regions’ baseline proportions were in the high and low level, respectively. The regional difference between the highest (80.8 % in the North East) and lowest (60.3 % in Northern Ireland) ACEIs prescription proportion was 20.5 %. However, the marginal difference between the highest and the lowest ACEIs prescription proportion declined over time from 20.5 % in April 2006 to 12 % in March 2012, i.e., the highest proportion was in East Midlands (75.8 %) while the lowest was in South East Coast (63.8 %). The results from the regression parsimonious models (Table 3, Additional file 2) indicated a wide variation in the policy impact on the ACEIs prescription proportion across various regions. Although there was no clear pattern of the policy impact, the policy generally seemed to have least influence in regions with the highest and lowest baseline ACEIs prescription proportions.

**Table 2:** Number of patients, antihypertensive prescriptions and baseline ACEIs prescription proportion in the 13 UK regions

Regions	Baseline ACEIs prescription proportion (%)	ACEIs prescription proportion (%) at the end of the study	Total number of prescriptions	Total number of patients
High baseline ACEIs prescription proportion (>74 %)				
North East	80.8	74.2	1,143,760	13,319
South East	74.7	71.6	3,865,373	53,299
Wales	74.4	74.1	6,185,924	63,467
East Midlands	74.3	75.8	1,364,324	22,370
Intermediate baselineACEIs prescription proportion (65 %-74 %)				
Yorkshire and the Humber	73.5	73.6	1,554,143	20,968
East of England	72.5	72.1	3,505,910	52,733
South Central	71.7	70.9	5,030,800	69,175
Scotland	72.2	70.8	3,760,187	62,610
West Midlands	69.5	68.5	3,764,019	52,920
North West	69.2	70.8	6,279,440	80,575
London	69.1	70.9	3,401,591	57,108
Low baseline ACEIs prescription proportion (<65 %)				
South East Coast	63.6	63.8	3,698,109	52,221
Northern Ireland	60.3	65.5	801,164	15,256

*ACEIs* Angiotensin converting enzyme inhibitors

**Table 3:** Segmented regression analysis on the monthly ACEIs prescription proportion in the 13 UK regions

Regions	β_1_^(a)^	β_2_^(b)^	β_3_^(c)^	β_4_^(d)^	β_5_^(e)^	β_6_^(f)^	β_7_^(g)^
High baseline ACEIs prescription proportion (>74 %)							
North East	−0.12 (−0.13, −0.11)	---	---	---	0.04 (0.02, 0.06)	---	---
South East	−0.06 (−0.70, −0.05)	−0.31 (−0.7, −0.07)	0.02 (0.01, 0.04)	---	---	---	---
Wales	---	---	---	---	---	---	---
East Midlands	−0.03 (−0.04, −0.16)	---	---	−0.64 (−1.1, −0.20)	0.16 (0.13, 0.19)	---	---
Intermediate baseline ACEIs prescription proportion (65 %-74 %)							
Yorkshire and the Humber	−0.08 (−0.09, −0.06)	---	0.20 (0.14, 0.24)	−1.80 (−0.26,-0.9)	---	---	---
East of England	−0.08 (−0.70, −0.05)	−0.40 (−0.7, −0.05)	0.02 (0.04, 0.03)	---	---	---	---
South Central	−0.01 (−0.2, −0.002)	−0.60 (−0.9, −0.40)	---	−0.47 (−0.7, −0.20)	---	---	---
Scotland	---	---	0.06 (0.04, 0.08)	−0.49 (−0.80, −0.10)	---	---	---
West Midlands	---	---	−0.03 (−0.04, −0.02)	---	---	---	---
North West	0.01 (0.02, 0.018)	−0.57 (−0.8, −0.33)	0.04 (0.03, 0.05)	---	---	---	---
London	---	0.40 (0.20, 0.60)	---	---	0.04 (0.03, 0.05)	---	---
Low baseline ACEIs prescription proportion (<65 %)							
South East Coast	0.013 (0.004, 0.02)	−0.42 (−0.7, −0.20)	−0.02 (−0.03,-0.002)	---	---	---	---
Northern Ireland	0.06 (0.05, 0.08)	---	---	---	0.06 (0.02, 0.09)	---	---

Regression coefficients (95 % confidence intervals) for the final model (the most parsimonious models); ^(a)^baseline trend; ^(b)^level change following BCBV policy; ^(c)^trend change following BCBV policy; ^(d)^level change following generic losartan availability; ^(e)^trend change following generic losartan availability; ^(f)^level change following generic perindopril availability; ^(g)^trend change following generic perindopril availability; −--: indicates insignificant estimates at 0.05 level, after stepwise backward elimination; ACEIs: Angiotensin converting enzyme inhibitors

### Monthly ACEIs/ARBs prescriptions’ cost

**Fig. 2:**
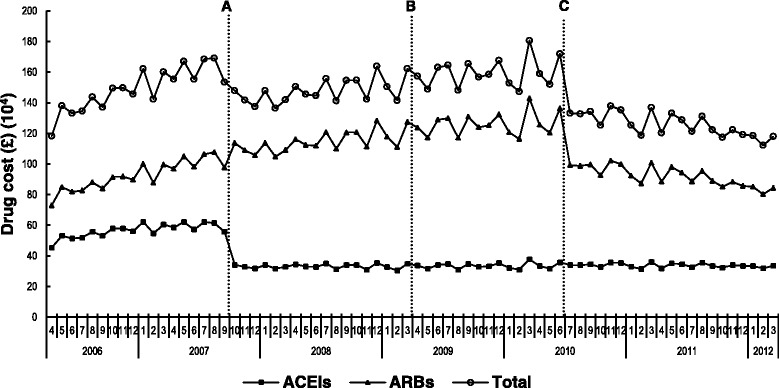
Monthly cost of total ACEIs/ARBs, ACEIs and ARBs from April 2006 to March 2012. **a** time point when generic perindopril became available (October 2007); **b**: time point when BCBV policy was implemented (April 2009); **c**: time point when generic losartan launched (July 2010); ACEIs: Angiotensin converting enzyme inhibitors; ARBs: Angiotensin receptor blockers

Overall, total ACEIs/ARBs cost decreased by only 0.3 % during the study period, from GB£1,182,656 in April 2006 to GB£1,178,674 in March 2012. However, there was a 26.1 % reduction in monthly ACEIs cost from GB£453,303 in April 2006 to GB£334,934 in March 2012, whereas the monthly ARBs cost increased by 15.7 %, from GB£729,353 to GB£843,740 (Fig. 2). The BCBV policy neither affected the level (β_2_) nor the trend (β_3_) of total ACEIs and ARBs, ACEIs or ARBs costs following its implementation in April 2009 (Table 4, Additional file 3).

**Table 4:** Segmented regression analysis on the monthly cost of ACEIs/ARBs, ACEIs and ARBs

Variables	β_1_^(a)^	β_2_^(b)^	β_3_^(c)^	β_4_^(d)^	β_5_^(e)^	β_6_^(f)^	β_7_^(g)^
ACEIs/ARBs	22072 (15546,28599)	---	---	−287538 (−368885,-206192)	−15970 (−21775,-10164)	−266656 (−349379,-183932)	−15019 (−22054,-7983)
ACEIs	6228.8 (4372, 8086)	---	---	---	---	−286941 (−308447,-265435)	−6094 (−7971,-4217)
ARBs	17030 (13449, 20611)	---	---	−298044 (−357163, −238924)	−15548 (−19712,-11384)	---	−9839 (−14560,-5118)

Regression coefficients (95 % confidence intervals) for the final model (the most parsimonious models); ^(a)^ baseline trend; ^(b)^ level change following BCBV policy; ^(c)^ trend change following BCBV policy; ^(d)^ level change following generic losartan availability; ^(e)^ trend change following generic losartan availability; ^(f)^ level change following generic perindopril availability; ^(g)^ trend change following generic perindopril availability; −--: indicates insignificant estimates at 0.05 level, after stepwise backward elimination; ACEIs: Angiotensin converting enzyme inhibitors; ARBs: Angiotensin receptor blockers

### Prediction of the potential cost saving of BCBV policy

A potential cost-saving of 23.9 % (GB£227,593) of the total ACEIs/ARBs costs would have been made in March 2012 if the ACEIs prescriptions’ proportion of 80 % of total RAS had been achieved, instead of the current value of 70.7 %, based on the total ACEIs/ARBs costs of GB£1,178,674 and the number of ACEIs prescriptions of 174,153 and ARBs prescriptions of 72,240 in March 2012. Applying this estimated 23.9 % cost-saving figure to England’s total ACEIs/ARBs spending in March 2012 of GB£2,220,548,343 would yield an equivalent cost-saving figure of GB£530,711,054.

The analysis of June 2014 PCA data of England indicated that if the 80 % BCBV target had been achieved in June 2014, instead of the current June 2014 value of 70.3 %, a potential cost-saving of GB£92,688,486 (8.7 %) of the total ACEIs/ARBs cost (GB£1.06 billion) would have been made in June 2014 from enhancing the prescribing efficiency of ACEIs and ARBs.

### Comparison with the national figures

Results from the HSCIC quarterly dispensing dataset analyses (Table 5, Additional file 4) showed a significant declining trend in the ACEIs prescriptions’ proportion prior to the BCBV policy implementation (β_1:_ -0.09 %, *p* < 0.001). Although the policy had no instant impact on the level (β_2_) of ACEIs prescriptions’ proportion, it resulted in a significant increase in the trend of the ACEI prescriptions’ proportion thereafter (β_3_: 0.1 %, *p* < 0.001). The results from the analysis of quarterly national dispensing data were consistent with that obtained from the CPRD dataset analysis (Table 5), which showed a significant decrease in the trend of ACEIs prescriptions proportion prior to the policy implementation (β_1_: −0.04 %, *p* < 0.001), no instant policy impact (β_2_), but a significant increase in the post-policy trend (β_3_: 0.04 %, *p* < 0.001).

**Table 5:** Segmented regression analysis on the quarterly number of antihypertensive prescriptions and ACEIs prescription proportion

Variables	β_1_^(a)^	β_2_^(b)^	β_3_^(c)^
Health and Social Care Information Centre (HSCIC) data			
ACEIs prescription proportion (%)	−0.09 (−0.10, −0.70)	---	0.10 (0.09, 0.13)
ACEIs	179422.8 (155964.2, 202881.5)	---	−88326.8 (−128278.1,-48375.6)
ARBs	87446.1 (77192.5, 97699.7)	---	−52359.4 (−69821.8, −34897.1)
Diuretics	---	---	---
CCBs	121943.8 (106805.8, 137081.8)	---	−40240.7 (−66021.5, 14459.9)
BBs	22331.1 (2253.2, 42409.0)	---	61787.6 (27593.9, 95983.2)
“Others”	15605.9 (12056.8, 19154.0)	---	−7041.9 (−13085.4, 998.4)
Clinical Practice Research Datalink (CPRD) data			
ACEIs prescription proportion (%)	−0.04 (−0.6, −0.02)	---	0.04 (0.01, 0.07)
ACEIs	15862.3 (14300.5, 17427.0)	---	−14529.7 (−17189.5, −11869.9)
ARBs	6993.5 (6377.5, 7609.4)	---	−6167.7 (−7216.6, −5118.6)
Diuretics	6414.9 (5325.2, 7504.5)	---	−8312.0 (−10167.6, −6456.2)
CCBs	11960.5 (10917.0, 13003.9)	---	−9576.0 (−11353.1, −7798.9)
BBs	1681.0 (948.6, 2413.4)	---	−1689.5 (−2936.7, −442.2)
“Others”	1765.0 (1572.7, 2002.3)	---	−1924.8 (−2328.9, −1520.6)

Regression coefficients (95 % confidence intervals) for the final model (the most parsimonious models); ^**(a)**:^baseline trend; ^**(b)**:^level change following BCBV policy; ^**(c)**:^trend change following BCBV policy; −-- : Indicates insignificant estimates at 0.05 level, after stepwise backward elimination; ACEIs: Angiotensin converting enzyme inhibitors; ARBs: Angiotensin receptor blockers; CCBs: Calcium channel blockers; BBs: Beta-blockers

**Fig. 3:**
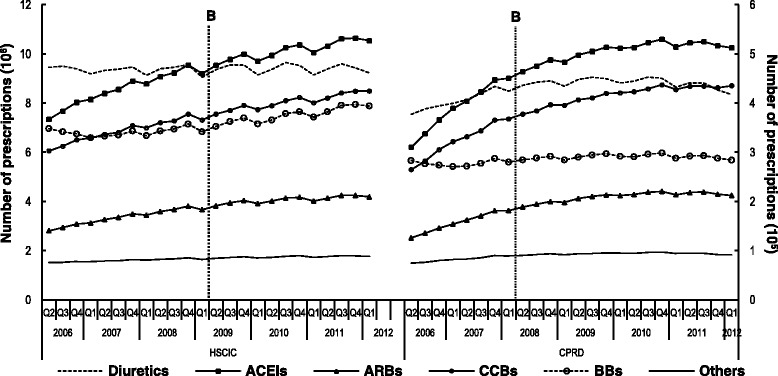
Quarterly number of antihypertensive prescriptions comparing HSCIC and CPRD dataset. **b**: time point when BCBV policy was implemented (April 2009); HSCIC: Health and Social Care Information Centre dataset, which contains whole dispensing data for all indications in England; CPRD: Clinical Practice Research Datalink, which contains prescription data for essential hypertension patients who registered in the practices included in CPRD; ACEIs: Angiotensin converting enzyme inhibitors; ARBs: Angiotensin receptor blockers; CCBs: Calcium channel blockers; BBs: Beta-blocker

The quarterly number of prescriptions of the six antihypertensive drug classes from the HSCIC dataset showed a significant pre-policy increasing trend (β_1_) in all antihypertensive drug classes, except for diuretics (Fig. 3). The policy implementation did not instantly impact on the level (β_2_) of the number of prescriptions for all antihypertensive classes; but significantly reduced the trend for almost all classes (β_3_) (Table 5). These results were comparable with the results derived from the CPRD database analysis (Table 5).

The results presented in Table 5 regarding CPRD could appear different from the results in Table 1 due to the difference in the level of analysed data since in the latter data were analysed at a monthly level; whereas in the former data were analysed at a quarterly level. Quarterly level analysis was performed on CPRD data in order to compare the results with the national data, which was only available as quarterly level data. In fact, the comparability of the results between the two data sets suggests the representativeness and generalisability of CPRD findings to the national UK population.

## Discussion

### Main findings

This study found that ACEIs prescribing as a proportion of RAS medicines was decreasing during the study period, and BCBV policy moderated the extent of this decreasing trend after its implementation as it was associated with a sustained monthly increase in ACEIs prescription proportion. However, although the increase was statistically significant, it was very small in magnitude, and it could be of no policy/clinical significance, especially the policy failed to achieve the expected target of 80 % ACEIs. We acknowledge though that the impact of BCBV policy varied among the 13 UK regions. The reduction in post-policy adjusted number of prescriptions for ACEIs/ARBs and the other antihypertensive classes (reference groups), imply that factors other than the BCBV policy could have contributed to the reduction in the number of prescriptions.

The policy had neither immediate nor sustained effect on total ACEIs/ARBs costs, ACEIs or ARBs costs individually. The results indicated that failure to achieve the 80 % BCBV target in March 2012 had led to miss potential cost-saving of 23.9 % of total ACEIs/ARBs cost. Importantly, although the potential cost-saving of efficient ACEIs/ARBs prescribing was moderated by the availability of generic ARBs, the analysis of June 2014 PCA data indicated that the failure of achieving the 80 % ACEIs target in June 2014 resulted in losing (8.7 %, GB£92,688,486) a potential cost-savings opportunity in the total ACEIs/ARBs costs (GB£1.06 billion). This potential opportunity observed in June 2014 was found despite four years after the availability of generic ARBs starting with generic losartan.

### Implementation of the BCBV policy

Generally, inappropriate policy measures and/or inadequate policy implementation are the two common causes underpinning any policy failure [46]. Since the BCBV measure of 80 % ACEIs prescription proportion was considered an achievable target [2], the lack of full implementation could be the main cause of the policy failure, especially the BCBV policy was not linked to any national financial incentives or legislation enforcements; consequently, general practitioners (GPs) may have been reluctant and/or less motivated to uptake the policy. However, this needs further investigation to provide learning lessons for future policies. The observed regional variation of the policy’s impact could reflect the possible variation of policy implementation as different regions may have been implementing various strategies to promote the policy uptake, and implementation balanced against other identified priorities.

This is consistent with the well-known fact that multiple measures are typically required to alter prescribing patterns effectively, given the complex nature of prescribing, and that a single measure could fail to achieve any significant change in prescribing behaviour [47–51]. It has been shown in other European countries that multiple intensive demand-side measures, e.g., combination of prescribing guidance, prescribing targets, financial incentives, and/or prescribing restrictions, are usually required to improve prescribing efficiency, including limiting the prescribing of patented ARBs vs. generic ACEIs [15, 17, 52, 53], and that countries with few measures, e.g., Portugal, failed to improve ARBs prescribing efficiency [15, 47].

Overall, the failure of linking BCBV policy with any national financial incentive or enforcement, as in QoF targets, or alternatively any universal PCT formulary and prescribing incentive scheme, may be the main contributor to the apparent poor implementation of the policy and hence its ineffectiveness. Consequently, specific measures will be required to enhance the implementation and uptake of this BCBV indicator, such as linking the target with financial incentives, and/or active switching programme, building on the previous experience with statins and proton pump inhibitors [54–57]. However, the active switching may disturb the patient-physician relationship and detriment therapeutic adherence; hence the policy should focus more on initiating eligible patient with ACEIs.

Instigating prescribing indicator for ACEIs vs. ARBs in the UK several years after the availability of ARBs was rather unique. Other countries, such as Sweden, instigated prescribing restrictions relegating ARBs to second line in 2008 several years after the availability of ARBs– so offers some comparisons. However, in Sweden, this was a prescribing restriction rather than a prescribing target; so greater impact in practice compared to no additional measures associated with BCBV since the prescribing restriction resulted in a 24 % reduction in the number of patients prescribed ARBs in the first four months after prescribing restrictions were introduced whilst increasing for ACEIs and CCBs, by 14 % and 12 %, respectively. The proportion initiated on ARBs prescribed an ACEI within 24 months prior to an ARB increased from 51 to 67 % [58].

### Other reasons for the policy failure

It could be argued that the observed lack of policy effectiveness merely reflects the fact that the encouragement of prescribing low cost generic ACEIs against patented ARBs was the subject of health authorities (HAs) in the UK some time before the launch of this BCBV policy, as seen in Scotland [59]. In Scotland, multiple measures were implemented between 2001 and 2007, including prescribing targets and financial incentives, and they resulted in a similar influence on limiting the prescribing of ARBs as those policies of prescribing restrictions coupled with financial incentives were implemented in Austria and Croatia [57].

Similarly, among PCTs in other parts of the UK, lots of local guidelines have been built based on NICE and NPC guidance as well as PCT activities [16–18]. Consequently, by the time this BCBV was launched in 2009, GPs and pharmaceutical advisers had turned their attention to other areas, hence, the limited impact of BCBV policy. A similar scenario was observed in Sweden, where the recent implementation of prescribing restrictions for patented statins had limited impact because these were introduced some six to seven years after pharmaceutical advisers and others working for the regions had been pushing for the increased prescribing of generic statins [60].

Although the previous HA and PCT activities could have played an important role in limiting ARBs utilisation, the declining ACEIs prescription proportion trend in the pre-policy period combined with its value being below the proposed target of 80 % should theoretically enhance the effectiveness of BCBV policy rather than impede its efficacy since there were opportunities for further improving the proportion of ACEIs prescriptions. Therefore, the activities of the HAs and PCTs in the UK in the previous years are unlikely to fully explain the lack of effectiveness of this BCBV indicator; although it was not possible to elicit what individual practice had actually done in terms of local initiatives to enhance ACEIs/ARBs prescribing efficiency following the introduction of this BCBV. We acknowledge this is a limitation of the paper. Furthermore, other factors such as GP’s prescribing preference and clinical issues related to switching from ARBs to ACEIs, and patients’ choice could not be ruled out as possible reasons for policy ineffectiveness.

### Impact of BCBV policy on number of prescriptions

The study results indicated a pre-policy increase in the trend of the monthly number of prescription of ACEIs, ARBs and CCBs, but a declining trend in those of diuretics, BBs and “Others”. The observed reduction in the trends of the last three groups could be related to their decreased roles in treating HT following the new NICE guidelines for treating HT [61] which specifically altered BBs’ place in therapy from first line treatment to fourth line of treatment and preferred CCBs over diuretics. This observed prescribing patterns were consistent with findings from another study [8] which found an increase in the prescribing of ACEIs, ARBs, and CCBs; while a reduction in prescribing of diuretics and BBs in patients with primary hypertension.

The reduction in post-policy adjusted number of prescriptions for all antihypertensive classes implies that some systematic factors, other than the BCBV policy may have contributed to the observed overall reduction. This hypothesis is considered plausible since the BCBV prescribing policy did not intend to affect the utilisation of ACEIs or ARBs, nor the utilisation of other antihypertensive classes, but instead it focused on the proportion of ACEIs vs. ARBs and encouraged GPs to prescribe cheaper, generic ACEIs, instead of expensive patented ARBs only if they made a decision to prescribe a drug that affects RAS.

Furthermore, one possible factor for the observed post-policy reduction in the prescribing trends of antihypertensive drugs could be an increase in hypertension diagnosis and registration after the introduction of QoF in April 2004, which incentivised GPs to produce a hypertension registry; hence, hypertension prevalence and antihypertensive drug prescriptions started to rise until it reached a plateau after years of the program introduction. The QoF-induced increase in hypertension registration reflected clearly on the annual increase of hypertension prevalence in the UK by about 2.8 %, from 12 % in 2005 to 13.4 % in 2009, in contrast to the 0.75 % increase from 13.4 % in 2009 to 13.6 % in 2011 [62].

### The impact of the launch of generic ACEIs/ARBs items on cost

This study found a 0.3 % decline in total ACEIs/ARBs cost during the study period, despite a 7.0 % increase in the adjusted total number of ACEIs/ARBs prescriptions. Although the adjusted number of prescriptions increased for both ACEIs (4.8 %) and ARBs (2.2 %), the cost implications were different. There was a 28.1 % decrease and 15.7 % increase of ACEIs and ARBs costs, respectively, largely attributable to the launch of generic perindopril and losartan in October 2007 and July 2010, respectively. Furthermore, the continuous decline in the cost of other generic ACEIs and losartan over time, prompted by the introduction of the ‘M’ (Manufacturer) and ‘W’ (Wholesaler) scheme in April 2005 [23], which aimed to increase the transparency in manufacturing and pricing of generics as well as any discounts offered to pharmacists by generic manufacturers, could also have contributed to the reduction in ACEIs/ARBs cost.

### Impact of generic ARBs on BCBV induced cost saving

Since ACEIs have started to almost lose their cost advantage over ARBs, driven by the availability of more low cost generic ARBs, it has been argued that ARBs should replace ACEIs as the first line therapy for hypertension treatment [63], which may indirectly suggest no further requirement for considering the BCBV indicator. However, this theoretical argument may not be translated into action in real life with NICE continuing to advocate both ACEIs and low cost ARBs as a first line medical treatment for hypertension.

Analysis of the England PCA data from March 2012 and June 2014 indicated only a small increase in losartan utilisation (8.9 % absolute increases over 27 month period), from 36.8 % of all ARBs items in March 2012 to 45.7 % in June 2014. Despite the fact that generic losartan had been listed in the Drug Tariff since July 2010, it is not surprising to see such a slow increase in losartan utilisation given the absence of national initiatives to improve ARBs prescribing efficiency as observed in Scotland [10], and NHS Bury in England until the intigation of multiple initiatives [59], apart from NICE and others advocating the prescribing of low cost ARBs alongside ACEIs before patented ARBs.

A classic case study conducted in NHS Bury in England [59] found that a combination of education, prescribing targets and financial incentives, implemented locally in March 2011 successfully increased losartan utilisation from 26 % of all ARBs in February 2011 to 65 % in October 2011; this again concluded that multiple initiatives were required to alter GPs’ prescribing behaviour in order to improve ARBs prescribing efficiency. Accordingly, due to the current lack of national multiple initiatives in the UK, it would be unlikely that losartan utilisation and ARBs prescribing efficiency would be improved further until more ARBs lose their patents, This would support why achieving the proposed BCBV target of 80 % ACEIs could still achieve potential cost-savings in the short term, even after years of losing the cost differential between ACEIs and ARBs.

Nevertheless, the importance of considering the efficient prescribing of RAS drugs in the UK, despite the availability of low cost generic ARBs, has been emphasised by retaining RAS drugs as one of the key therapeutic topics for 2015 to support the QIPP framework [64], as it is not known how fast or far the price of various generic ARBs would fall after they came off patent and it may take probably some years before several ARBs become available at similar costs to the current generic ACEIs costs [65]. Indeed, the latter fact can be clearly observed if we compared the monthly cost of the typical maintenance dose of the most commonly prescribed ACEIs ramipril to that of generic ARBs [66]. For example, even after more than four years of its launch as generic in July 2010, the cost of 28-day treatment of losartan (100 mg) is still about 14 % higher than that of ramipril (10 mg) (GB£1.12 vs.GB£1.27). Likewise, the cost of 28-day candesartan (16 mg), which became off patent in April 2012, is about 75.9 % higher in comparison to ramipril (GB£1.12 vs.GB £1.97) despite more than two years since its availability as generic.

### Implications of the study

The rationale for the BCBV policy was to ensure efficient use of healthcare resources without affecting the quality of care [29]. This study found that the policy has no impact on the number and cost of ACEIs/ARBs prescriptions, despite a small impact on ACEIs prescriptions as an overall proportion of RAS drugs. However, the opportunity of a potential saving is still possible via enhancement of efficient ACEIs/ARBs prescribing in NHS primary care practices, even after narrowing the cost differential between ACEIs and ARBs. These results suggest the ongoing necessity for reinforcing the BCBV indicator through the introduction of national and local measures to enhance its implementation and uptake. Nevertheless, the opportunity cost of introducing these measures must also be considered alongside those required for other high priority areas for efficiency improvement.

To appropriately reinforce the policy, reasons underpinning such failure need further exploration to develop initiatives to improve the policy effectiveness, building on existing experience from other European countries, such as qualitative research to explore physicians’ perceptions of the BCBV policy and the possible causes for BCBV failure.

### Strengths and limitations

To our knowledge, this is the first study to quantify and evaluate the impact of the BCBV policy on ACEIs/ARBs utilisation, using a quasi-experimental design on a large, representative, and high quality dataset. The interrupted segmented regression analysis ensured that post-intervention changes were not solely a continuation of the long-term trend, as the analysis controlled for existing, pre-intervention trend [35]. In addition, the findings of this study were consistent with the analysis results from another dataset, indicating the explicitly of study design and methodology, with the results are reflective to the national figures.

However, in this secondary data analysis, it was not possible to determine whether the prescribed drugs were purely prescribed for hypertension or for other indications such as heart failure, which may overestimate the observed antihypertensive drug utilisation quantity. Although it was not possible to identify an area free of policy as a reference group to separate the true policy impact from other policies, by adopting the alternative approach of using other antihypertensive drugs as reference groups and considering the impact for the potential confounders (launch of generic losartan and generic perindopril), it possibly allowed the estimation of the true intervention effect.

Using 2012 price unit instead of the current up to date price might have biased the cost-saving impact of the BCBV indicator as the current prices, especially those of ARBs, might have been lowered following the increasing availability of ARBs as generics as well as potentially lowering prices of existing generic ARBs. However, using June 2014 dispensing data for estimating the cost-saving impact of the BCBV indicator whilst accounting for availability of several generic ARBs would have potentially minimised that bias and provided an approximate picture of the actual impact of BCBV. Finally, we acknowledge that we did not undertake simultaneous qualitative research among PCTs to ascertain what possible explanation there could be for no appreciable difference in the prescribing of ACEIs vs. ARBs despite potentially appreciable cost savings. However, this will be included into future research.

## Conclusions

The BCBV policy did not change the overall ACEIs/ARBs utilisation patterns in patients with primary hypertension. Although the BCBV policy was associated with a small, significant increase in ACEIs prescription proportion, there was no beneficial cost consequence. Intensive and specific measures would be required to enhance the policy implementation and uptake, given the existing opportunity for potential saving by further improving the efficient prescribing of ACEIs/ARBs despite the availability of low cost generic ARBs. However, this must be balanced against other priority areas for improving prescribing quality and efficiency. This study represents a case study of failed and ineffective policy promoted probably by its poor implementation. This could be a key learning point for other healthcare authorities to emphasise requirements for effective implementation strategies as well as the principle policy itself. Further research is needed to explore the potential factors underpinning the policy ineffectiveness, and identify the most effective strategies to improve future policy effectiveness.

## Additional files

Additional file 1:
**Segmented regression analysis, with all the parameter estimates, on monthly adjusted number of prescription of the six antihypertensive drug classes.** (DOCX 36 kb) Additional file 2:
**Segmented regression analysis, with all the parameter estimates, on the monthly ACEIs prescription proportion in the 13 UK regions.** (DOCX 37 kb) Additional file 3:
**Segmented regression analysis, with all the parameter estimates, on the monthly cost of ACEIs/ARBs, ACEIs and ARBs.** (DOCX 35 kb) Additional file 4:
**Segmented regression analysis, with all the parameter estimates, on the quarterly number of antihypertensive prescriptions and ACEIs prescription proportion.** (DOCX 35 kb)

## Abbreviations

ACEIs: Angiotensin-Converting Enzyme Inhibitors
ARBs: Angiotensin Receptor Blockers
BBs: Beta-Blockers
BCBV: Better Care Better Value
BNF: British National Formulary
CCBs: Calcium-Channel Blockers
CPRD: Clinical Practice Research Datalink
GPs: General Practitioners
HAs: Health Authorities
HSCIC: Health and Social Care Information Centre
NAO: National Audit Office
NHS: National Health Service
NICE: National Institute for Health and Care Excellence
NPC: National Prescribing Centre
OFT: Office of Fair Trading
PCA: Prescription Cost Analysis
PCTs: Primary Care Trusts
QIPP: Quality, Innovation, Productivity and Prevention
QoF: Quality and Outcomes Framework
RAS: Renin-Angiotensin System
