# Supplementary material for: The impact of the ‘Better Care Better Value’ prescribing policy on the utilisation of angiotensin-converting enzyme inhibitors and angiotensin receptor blockers for treating hypertension in the UK primary care setting: longitudinal quasi-experimental design
Source: BMC Health Serv Res. 2015 Sep 10;15:367. doi: 10.1186/s12913-015-1013-y (PMC4566432; doi:10.1186/s12913-015-1013-y)
Supplement: Additional file 1: — Segmented regression analysis, with all the parameter estimates, on monthly adjusted number of prescription of the six antihypertensive drug classes. (DOCX 36 kb) [file 12913_2015_1013_MOESM1_ESM.docx]

Additional file 1. Segmented regression analysis, with all the parameter estimates, on monthly adjusted number of prescription of the six antihypertensive drug classes

| **Variables** | **β_1_ ^(^**^a^**^)^** | **β_2_** ^(b)^ | **β_3_** ^(c)^ | **β_4_** ^(d)^ | **β_5_** ^(e)^ | **β_6_** ^(f)^ | **β_7_** ^(g)^ |
| --- | --- | --- | --- | --- | --- | --- | --- |
| **ACEIs prescription**  **proportion (%)** | **-0.02**  **(-0.2, -0.01)** | **-0.30**  **(-0.44, -0.16)** | **0.013**  **(0.007, 0.02)** | -0.11  (-0.32, 0.09) | 0.01  (-0.02, 0.04) | -0.14  (-0.32, 0.04) | -0.03  (-0.04, 0.008) |
| **Adjusted number of**  **prescriptions** | | | | | | | |
| ACEIs | **135.7**  **(117.8, 153.6)** | -415.8  (-1061.4, 229.8) | **-149.9**  **(-181.4, -118.4)** | 662.1  (-161.6, 1485.7) | -8.8  (-145.6, 128.0) | -183.1  (-983.2, 617.0) | -84.6  (-161.5, 70.6) |
| ARBs | **65.9**  **(58.7, 72.0)** | -178.1  (-553.7, 197.5) | **-67.2**  **(-79.8, -54.6)** | 334.0  (-48.5, 716.5) | -17.4  (-50.5, 15.7) | -18.3  (-355.8, 319.3_ | -59.7  (-92.2, 27.3) |
| Diuretics | **-55.4**  **(-61.9, -48.9)** | 448.1  (-81.5, 977.6) | -30.2  (-65.6, 5.1) | 297.1  (-206.9, 801.1) | 50.7  (-60.9, 162.2) | 209.8  (-535.5, 975.1) | 33.3  (-39.3, 105.9) |
| CCBs | **90.4**  **(77.0, 103.9)** | -220.8  (-708.6, 267.1) | **-77.4**  **(-101.1, -53.8)** | 494.5  (-124.1, 1113.1) | 4.2  (-99.2, 107.6) | -54.8  (-712.7, 603.1) | -98.9  (-162.2, 35.7) |
| BBs | **-102.7**  **(-119.0, -86.5)** | 227.6  (-403.4, 858.6) | **-75.3**  **(-98.3, -52.4)** | 194.0  (-415.9, 803.9) | 11.2  (-90.4, 112.8) | 565.3  (-31.1, 1099.5) | 159.3  (-107.9, 210.7) |
| “Others” | **-3.0**  **(-4.9, -1.0)** | 30.1  (-76.9, 137.2) | **-15.6**  **(-21.1, -10.1)** | 170.4  (-35.2, 305.6) | -1.8  (-24.5, 20.8) | 76.5  (-77.3, 230.4) | -2.0  (-16.8, 12.8) |

**(Note)** **^(a)^** baseline trend; **^(b)^** level change following BCBV policy; **^(c)^** trend change following BCBV policy; **^(d)^** level change following generic losartan availability; **^(e)^** trend change following generic losartan availability; **^(f)^** level change following generic perindopril availability; ^(g)^ trend change following generic perindopril availability; **Bold**: indicates the significant parameter estimates from the most parsimonious models; ACEIs: Angiotensin converting enzyme inhibitors; ARBs: Angiotensin receptor blockers; CCBs: Calcium channel blockers; BBs: Beta-blocker.
