# Supplementary material for: The impact of the ‘Better Care Better Value’ prescribing policy on the utilisation of angiotensin-converting enzyme inhibitors and angiotensin receptor blockers for treating hypertension in the UK primary care setting: longitudinal quasi-experimental design
Source: BMC Health Serv Res. 2015 Sep 10;15:367. doi: 10.1186/s12913-015-1013-y (PMC4566432; doi:10.1186/s12913-015-1013-y)
Supplement: Additional file 2: — Segmented regression analysis, with all the parameter estimates, on the monthly ACEIs prescription proportion in the 13 UK regions. (DOCX 37 kb) [file 12913_2015_1013_MOESM2_ESM.docx]

Appendix 1

Additional file 2. Segmented regression analysis, with all the parameter estimates, on the monthly ACEIs prescription proportion in the 12 UK regions

| **Regions** | **β_1_ ^(^**^a^**^)^** | **β_2_** ^(b)^ | **β_3_** ^(c)^ | **β_4_** ^(d)^ | **β_5_** ^(e)^ | **β_6_** ^(f)^ | **β_7_** ^(g)^ |
| --- | --- | --- | --- | --- | --- | --- | --- |
| ***High baseline ACEIs prescription***  ***proportion (>74%)*** | | | | | | | |
| North East | **-0.12**  **(-0.13, -0.11)** | 0.54  (-0.10, 1.18) | 0.04  (-0.05, 0.12) | -0.05  (-0.61, 0.52) | **0.04**  **(0.02, 0.06)** | -0.78  (-1.28, 0.20) | -0.10  (-0.16, 0.05) |
| South East | **-0.06**  **(-0.70, -0.05)** | **-0.31**  **(-0.7, -0.07)** | **0.02**  **(0.01, 0.04)** | 0.10  (-0.34, 0.36) | 0.009  (-0.04, 0.06) | 0.21  (-0.13, 0.55) | -0.05  (-0.08, 0.02) |
| Wales | 0.01  (-0.05, 0.03) | -0.06  (-0.40, 0.29) | -0.001  (-0.05, 0.04) | -0.10  (-0.40, 0.21) | 0.003  (-0.04, 0.05) | -0.20  (-0.49, 0.09) | -0.03  (-0.05, 0.002) |
| East Midlands | **-0.03**  **(-0.04, -0.16)** | -0.16  (-0.92, 0.60) | 0.04  (-0.06, 0.14) | **-0.64**  **(-1.1, -0.20)** | **0.16**  **(0.13, 0.19)** | -0.12  (-0.76, 0.52) | -0.003  (-0.065, 0.06) |
| ***Intermediate baseline ACEIs prescription***  ***proportion (65%-74%)*** | | | | | | | |
| Yorkshire and the Hum | **-0.08**  **(-0.09, -0.06)** | 0.20  (-0.84, 1.23) | **0.20**  **(0.14, 0.24)** | **-1.80**  **(-0.26,-0.9)** | -1.54  (-2.54, 0.54) | -0.04  (-1.01, 0.91) | 0.09  (-0.01, 0.19) |
| East of England | **-0.08**  **(-0.70, -0.05)** | **-0.40**  **(-0.7, -0.05)** | **0.02**  **(0.04, 0.03)** | -0.01  (-0.42, 0.39) | -0.05  (-0.11, 0.04) | 0.08  (-0.31, 0.47) | -0.09  (-0.13, 0.06) |
| South Central | **-0.01**  **(-0.2, -0.002)** | **-0.60**  **(-0.9, -0.40)** | 0.03  (-0.2, 0.09) | **-0.47**  **(-0.7, -0.20)** | -0.0002  (-0.05, 0.04) | -0.03  (-0.36, 0.31) | -0.06  (-0.09, 0.03) |
| West Midlands | 0.02  (-0.01, 0.04) | 0.18  (-0.25, 0.60) | **-0.03**  **(-0.04, -0.02)** | 0.36  (-0.20, 0.74) | 0.07  (-0.002, 0.11) | -0.33  (-0.69, 0.03) | 0.005  (-0.03, 0.04) |
| North West | **0.01**  **(0.02, 0.018)** | **-0.57**  **(-0.8, -0.33)** | **0.04**  **(0.03, 0.05)** | 0.17  (-0.12, 0.47) | -0.01  (-0.05, 0.04) | -0.42  (-0.70, 0.14) | -0.06  (-0.08, 0.03) |
| London | -0.01  (-0.04, 0.02) | **0.40**  **(0.20, 0.60)** | -0.01  (-0.07, 0.05) | 0.03  (-0.40, 0.45) | 0.04  (0.03, 0.05) | -0.13  (-0.53, 0.27) | 0.03  (-0.004, 0.07) |
| ***Low baseline ACEIs prescription***  ***proportion (<65%)*** | | | | | | | |
| South East Coast | **0.013**  **(0.004, 0.02)** | **-0.42**  **(-0.7, -0.20)** | **-0.02**  **(-0.03,-0.002)** | 0.05  (-0.35, 0.45) | 0.01  (-0.04, 0.07) | -0.31  (-0.70, 0.07) | -0.02  (-0.05, 0.02) |
| Northern Ireland | **0.06**  **(0.05, 0.08)** | -0.26  (-1.27, 0.80) | 0.01  (-0.13, 0.14) | -0.62  (-1.51, 0.30) | **0.06**  **(0.02, 0.09)** | 0.80  (-0.10, 1.61) | 0.11  (-0.03, 0.19) |

**(Note)** **^(a)^** baseline trend; **^(b)^** level change following BCBV policy; **^(c)^** trend change following BCBV policy; **^(d)^** level change following generic losartan availability; **^(e)^** trend change following generic losartan availability; **^(f)^** level change following generic perindopril availability; ^(g)^ trend change following generic perindopril availability; **Bold**: indicates the significant parameter estimates from the most parsimonious models.
