# Supplementary material for: The impact of the ‘Better Care Better Value’ prescribing policy on the utilisation of angiotensin-converting enzyme inhibitors and angiotensin receptor blockers for treating hypertension in the UK primary care setting: longitudinal quasi-experimental design
Source: BMC Health Serv Res. 2015 Sep 10;15:367. doi: 10.1186/s12913-015-1013-y (PMC4566432; doi:10.1186/s12913-015-1013-y)
Supplement: Additional file 3: — Segmented regression analysis, with all the parameter estimates, on the monthly cost of ACEIs/ARBs, ACEIs and ARBs. (DOCX 35 kb) [file 12913_2015_1013_MOESM3_ESM.docx]

Appendix 1, Appendix 2

Additional file 3. Segmented regression analysis, with all the parameter estimates, on the monthly cost of ACEIs/ARBs, ACEIs and ARBs

| **Variables** | **β_1_ ^(^**^a^**^)^** | **β_2_** ^(b)^ | **β_3_** ^(c)^ | **β_4_** ^(d)^ | **β_5_** ^(e)^ | **β_6_** ^(f)^ | **β_7_** ^(g)^ |
| --- | --- | --- | --- | --- | --- | --- | --- |
| ACEIs/ARBs | **22072**  **(15546,28599)** | 42834.2  (-94836.7, 180505.1) | -9966.8  (-23390.6, 8456.9) | **-287538**  **(-368885,-206192)** | **-15970**  **(-21775,-10164)** | **-266656**  **(-349379,-183932)** | **-15019**  **(-22054,-7983)** |
| ACEIs | **6228.8**  **(4372, 8086)** | 4688.3  (-27680.7, 37057.2) | -845.7  (-5177.5, 3486.1) | 20232.7  (-8432.8, 48889.3) | 175.8  (-3896.8, 4248.4) | **-286941**  **(-308447,-265435)** | **-6094**  **(-7971,-4217)** |
| ARBs | **17030**  **(13449, 20611)** | 38106.7  (-70623.5, 146836.9) | -9081.0  (-23631.9, 5469.8) | **-298044**  **(-357163, -238924)** | **-15548**  **(-19712,-11384)** | 13227.6  (-78832.2, 105287.3) | **-9839**  **(-14560,-5118)** |

**(Note)** **^(a)^** baseline trend; **^(b)^** level change following BCBV policy; **^(c)^** trend change following BCBV policy; **^(d)^** level change following generic losartan availability; **^(e)^** trend change following generic losartan availability; **^(f)^** level change following generic perindopril availability; ^(g)^ trend change following generic perindopril availability; **Bold**: indicates the significant parameter estimates from the most parsimonious models; ACEIs: Angiotensin converting enzyme inhibitors; ARBs: Angiotensin receptor blockers; CCBs: Calcium channel blockers; BBs: Beta-blocker.
