# Supplementary material for: The impact of the ‘Better Care Better Value’ prescribing policy on the utilisation of angiotensin-converting enzyme inhibitors and angiotensin receptor blockers for treating hypertension in the UK primary care setting: longitudinal quasi-experimental design
Source: BMC Health Serv Res. 2015 Sep 10;15:367. doi: 10.1186/s12913-015-1013-y (PMC4566432; doi:10.1186/s12913-015-1013-y)
Supplement: Additional file 4: — Segmented regression analysis, with all the parameter estimates, on the quarterly number of antihypertensive prescriptions and ACEIs prescription proportion. (DOCX 35 kb) [file 12913_2015_1013_MOESM4_ESM.docx]

Appendix 1, Appendix 2

Additional file 4. Segmented regression analysis, with all the parameter estimates, on the quarterly number of antihypertensive prescriptions and ACEIs prescription proportion

| **Variables** | **β_1_ ^(^**^a^**^)^** | **β_2_** ^(b)^ | **β_3_** ^(c)^ |
| --- | --- | --- | --- |
| ***Health and Social Care***  ***Information Centre (HSCIC) data*** | | | |
| ACEIs prescription proportion (%) | **-0.09 (-0.10, -0.70)** | -0.23 (-0.36, 0.09) | **0.10 (0.09, 0.13)** |
| ACEIs | **179422.8 (155964.2, 202881.5)** | -33538.6 (-318837.8, 251760.6) | **-88326.8 (-128278.1,-48375.6)** |
| ARBs | **87446.1 (77192.5, 97699.7)** | 38294.2 (-85311.3, 161899.6) | **-52359.4 (-69821.8, -34897.1)** |
| Diuretics | -10080.0 (-39682.7, 19855.8) | 153403.8 (-137145.4, 443953.0) | 3490.3 (-38374.3, 45354.8) |
| CCBs | **121943.8 (106805.8, 137081.8)** | -15604.9 (-66625.1, 13532.3) | **-40240.7 (-66021.5, 14459.9)** |
| BBs | **22331.1 (2253.2, 42409.0)** | 115778.6 (-122734.2, 354291.4) | **61787.6 (27593.9, 95983.2)** |
| “Others” | **15605.9 (12056.8, 19154.0)** | 13730.9 (-29014.5, 56476.2) | **-7041.9 (-13085.4, 998.4)** |
| ***Clinical Practice Research***  ***Datalink (CPRD) data*** | | | |
| ACEIs prescription proportion (%) | **-0.04 (-0.6, -0.02)** | -0.33 (-0.55, 0.10) | **0.04 (0.01, 0.07)** |
| ACEIs | **15862.3 (14300.5, 17427.0)** | -1403.8 (-20414.8, 17607.3) | **-14529.7 (-17189.5, -11869.9)** |
| ARBs | **6993.5 (6377.5, 7609.4)** | 2849.2 (-4534.5, 10232.9) | **-6167.7 (-7216.6, -5118.6)** |
| Diuretics | **6414.9 (5325.2, 7504.5)** | 6602.6 (-6307.3, 19512.4) | **-8312.0 (-10167.6, -6456.2)** |
| CCBs | **11960.5 (10917.0, 13003.9)** | -596.1 (-13302.4, 12110.2) | **-9576.0 (-11353.1, -7798.9)** |
| BBs | **1681.0 (948.6, 2413.4)** | 8017.2 (-81.5, 16115.9) | **-1689.5 (-2936.7, -442.2)** |
| “Others” | **1765.0 (1572.7, 2002.3)** | 608.8 (-2267.5, 3485.1) | **-1924.8 (-2328.9, -1520.6)** |

**(Note)** **^(a)^**^:^ baseline trend; **^(b)^**^:^ level change following BCBV policy; **^(c)^**^:^ trend change following BCBV policy; **Bold**: indicates the significant parameter estimates from the most parsimonious models; ACEIs: Angiotensin converting enzyme inhibitors; ARBs: Angiotensin receptor blockers; CCBs: Calcium channel blockers; BBs: Beta-blockers.
